# Niacin Ameliorates Lipid Disturbances due to Glucocorticoid Administration in Rats

**Published:** 2012

**Authors:** Niloofar Safaei, Tahoora Shomali, Mahnaz Taherianfard

**Affiliations:** 1 *Division of Physiology, Department of Basic Sciences, School of Veterinary Medicine, Shiraz University, Shiraz, Iran *; 2 *Division of Pharmacology and Toxicology, Department of Basic Sciences, School of Veterinary Medicine, Shiraz University, Shiraz, Iran*

**Keywords:** Dyslipidemia, Fatty Liver, Glucocorticoids, Niacin, Rats

## Abstract

**Objective(s):**

This study was conducted to evaluate the effects of niacin on glucocorticoid-induced dyslipidemia and fatty liver in rats.

**Materials and Methods:**

Twenty four adult male rats were divided randomly into four equal groups: 1- normal saline (control), 2- dexamathasone 0.125 mg/kg/day, i.m., 3- dexamathasone + niacin 200 mg/kg/day, oral gavages, 4- niacin. After 2 weeks, serum total cholesterol, triglycerides, HDL-c, LDL-c and VLDL-c concentrations were assayed and liver sections examined for fatty liver changes. Data were analyzed by ANOVA method and *P*< 0.05 was considered significant.

**Results:**

Dexamethasone increased all lipid parameters as compared to control (*P*< 0.05). Lipid parameters in group 3, were lower than group 2 (*P*< 0.05) except for HDL-c which remained statistically the same. Moderate fatty liver changes were observed in one third of rats in dexamethasone group. Rats in groups 1, 3 and 4 had no sign of fatty changes.

**Conclusion:**

Niacin positively affects glucocorticoid-induced dyslipidemia and fatty liver changes in rats.

## Introduction

Glucocorticoids (GCs) have profound anti-inflammatory and immunosuppressive properties that are critical for the treatment of a variety of diseases such as rheumatoid arthritis, cerebral edema, allergic reactions, *etc.* (1). However, GC use, especially at high doses or for prolonged period, has been associated with many potential adverse effects, such as muscle wasting, diabetes, hypertension and osteoporosis (2). Moreover, GCs can elevate plasma lipids in humans (3) and induce dyslipidemia in laboratory animals (4); in a way that these agents have been recognized as a secondary cause of dyslipidemia. The fact that pharmacologic doses of GCs can affect plasma lipid levels has been known for decades. It has been extensively shown that GC administration is associated with serum lipid disturbances including elevations in total cholesterol, triglycerides, LDL-c and HDL-c in humans (5) as well as laboratory animals (4). Moreover, dyslipidemia may be present in humans with Cushing’s syndrome (6). On the other hand, GCs are known to contribute to fatty liver production (7) and a high prevalence (up to 20%) of fatty liver has been reported in people afflicted with Cushing’s syndrome (6). 

Since its initial elucidation, more than 50 years ago in a land mark study by Altshul *et al,* niacin (nicotinic acid or vitamin B_3_) administration has shown beneficial effects on traditional lipoprotein fractions. This agent has recently attracted renewed interest; first because it is currently the most potent drug increasing HDL-c and, secondly, because it has been found to induce regression of atherosclerosis (8). However, effects of niacin on GC-induced lipid disturbances have not been clarified. Regarding the particular feature of GC-induced dyslipidemia (high HDL-c levels), The present study was conducted to evaluate effects of niacin on dyslipidemia and fatty changes of liver due to dexamethasone, a potent GC receptor agonist with insignificant mineralocorticoid receptor activity, in rats as a frequently used animal model for dyslipidemic conditions.

## Materials and Methods

***Animals and experimental design***

Twenty four adult male Sprague-Dawley rats with a mean body weight of 230 g were purchased from animal house of Shiraz Medical University, Shiraz, Iran. Rats were acclimatized to the ambient conditions (temperature about 23 ˚C and a 12 hr/12 hr, light/dark cycle) for one week before the beginning of the experiment. Then the animals were randomly allocated into four equal groups (six animals each) and treated for two weeks as follows: group one (control) received normal saline daily by intramuscular injections in a volume equal to the dexamethasone administered to the rat with the same weight; group two (dexamethasone) were given 0.125 mg/kg dexamethasone sodium phosphate (Darou Pakhsh Pharma Chem Co, Tehran, Iran) daily by intramuscular injections; group three (dexamethasone+niacin) were treated with 0.125 mg/kg dexamethasone daily by intramuscular injections along with 200 mg/kg niacin (Novin Kavosh Mamtir Co., Tehran, Iran) daily by oral gavages and group four (niacin) were given 200 mg/kg niacin daily by oral gavages. Dosage of dexamethasone and niacin were chosen according to the works accomplished by Bagdade *et al* 1976 and Barboriak and Meade 1971, respectively (9, 10). 

Animals had free access to tap water and standard rat chow diet prepared by Razi Vaccine and Serum Research Institute, . All animals were weighed daily during the experiment.

All Procedures used were in accordance with Institutional Ethical Guidelines of School of Veterinary Medicine, Shiraz University, for care and use of laboratory animals in experiments.

***Sample collection and determination of serum lipid parameters***

At the end of the experimental period, after an overnight starvation, blood samples were collected from all animals under chloroform anesthesia by cardiac puncture. Harvested sera were stored in -70 ˚C until use. Total cholesterol, triglycerides, LDL-c, HDL-c and VLDL-c concentrations were measured in collected sera. Total cholesterol and triglycerides were assayed by CHOD-PAP and GPO-PAP colorimetric methods, respectively. Enzymatic methods were used for determination of LDL-c and HDL-c concentrations. VLDL-c concentration was calculated as 0.2 triglycerides concentration. Kits were provided by Parsazmun Company, . 

**Figure 1:**
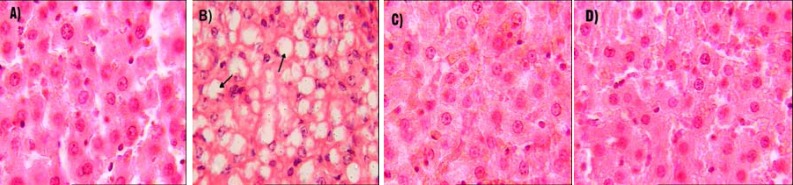
Representative liver sections from rats in different experimental groups (n= 6), stained with hematoxylin and eosin. Original magnification was x40

No sign of fatty changes observed in control (A); dexamethasone+ niacin (C) or niacin (D) treated rats. Cytoplasmic lipid vacuoles (arrows) were present in hepatocytes from one third of rats treated with dexamethasone (B)

***Histological examination of liver***

After blood collection, animals were killed by deepening anesthesia and samples of liver were fixed in 10% buffered formaldehyde solution for 24 hr and then 4% buffered formaldehyde solution. After routine histological processes, 5 μm thick sections were made and stained with hematoxylin and eosin to be examined under light microscope.

***Statistical analysis***

Data presented as mean±SD. Data analysis was carried out by using one-way ANOVA and Tukey’s as the *post hoc* test (SPSS 11.5 software for windows). Differences were considered significant at *P*< 0.05.

## Results

***Body weight and serum lipid parameters***

No significant difference was observed in body weight of rats from different groups during the experiment (data not shown). 

Dexamethasone treatment significantly increased total cholesterol level as compared to control group (*P*< 0.001), while rats treated with dexamethasone+niacin had statistically the same level of total cholesterol with control group (*P*> 0.05) which was significantly lower than rats treated with dexamethasone alone (*P*< 0.001). Niacin administration decreased total cholesterol concentration to the level even lower than control group (*P*= 0.009). Rats treated with dexamethasone had significantly higher serum triglycerides level in comparison with control group (*P*< 0.001). Although administration of niacin along with dexamethasone significantly decreased triglycerides concentration as compared to dexamethasone treated rats (*P*< 0.001), this parameters did not reach the control level (*P*< 0.001). Dexamethasone administration significantly increased LDL-c concentration as compared to control (*P*< 0.001). Concomitant administration of niacin with dexamethasone had a profound effect on LDL-c level; where this parameters decreased to a level even lower than control group (*P*< 0.001). Niacin treated rats had the lowest LDL-c concentration as compared to all other groups (*P*< 0.001). As it was expected, all treatments significantly increased serum HDL-c level as compared to control (*P*= 0.03 for dexamethasone group and *P*< 0.001 for dexamethasone+niacin and niacin treated groups) and the highest level of HDL-c was observed in niacin treated rats. Serum HDL-c level of rats in groups two and three was statistically the same (*P*> 0.05). VLDL-c level in rats treated with dexamethasone was about two times higher than control (*P*< 0.001). Rats treated with dexamethasone+niacin or niacin had significantly lower VLDL-c level in comparison with rats treated with dexamethasone (*P*< 0.001 for both groups). Data presented as mean±SD in Table 1.

**Table 1:** Serum lipid parameters presented as mean ± SD in different groups (n= 6) at the end of the experiment

	Parameters	Control	Dexamethasone	Dexamethasone + Niacin	Niacin
Groups					
Total cholesterol (mg/dl)		56.50±2.88^#^	79.00±3.03^*^	56.67±2.16^#^	51.00±1.58^*, #^
Triglycerides (mg/dl)		38.66±2.16^#^	72.83±2.63^*^	62.50±3.08^*, #^	51.80±2.38^*, #^
LDL-c (mg/dl)		21.31±1.36^#^	33.85±1.53^*^	11.80±1.16^*, #^	4.14±0.40^*, #^
HDL-c (mg/dl)		28.00±2.19^#^	30.78±1.64^*^	32.51±0.78^*^	38.26±1.27^*, #^
VLDL-c (mg/dl)		7.73±0.43^#^	14.56±0.52^*^	12.50±0.61^*, #^	10.36±0.47^*, #^

*and # signs are used to demonstrate significant differences with control and dexamethasone groups, respectively (*P*< 0.05)

***Histological examination of liver***

Moderate fatty change observed in liver of one third of rats from dexamethasone group. Lipid vacuoles were present in cytoplasm of hepatocytes especially in periacinar area of the liver. No sign of fatty change was observed in the liver of rats from control, dexamethasone+niacin or niacin groups (Figure 1).

## Discussion

GCs have been used in an attempt to treat practically every malady that afflicts man or animal. These agents have effects on virtually every cell type and system in mammals and are among the most widely prescribed drugs in the world. Unfortunately, the development of major metabolic side effects remains as the key limitation for long-term or high-dose use of GCs (2). Mechanisms whereby GCs lead to dyslipidemia could be summarized as direct increase in production of HDL in the liver, impaired catabolism of LDL, increase in the activity of lipoprotein lipase and subsequent increase in LDL and HDL_2_ levels and finally indirect increase in LDL and VLDL levels due to increased plasma insulin (5).

Chronically elevated GC levels have been intrinsically tied to fatty liver development. Although GCs are known to contribute to fatty liver production through a combination of increased fatty acid synthesis and decreased fatty acid β oxidation (7); the metabolic and molecular mechanisms of GC-dependent fatty liver development still remain largely elusive (11). 

Niacin has profound and unique effects on lipid metabolism and is thus referred to as a "broad spectrum anti-hyperlipidemic drug". This agent has predictable effects on all traditionally measured lipid parameters and remains the most potent agent for raising HDL. Niacin not only increases HDL-c and ApoA-I, but reduces LDL-c and triglycerides. The mechanism, by which niacin causes these beneficial effects on the lipid panel, continues to be investigated (12).

 It is currently believed that the main way in which niacin raises HDL is through reduced catabolism. Niacin selectively increases ApoA-I–containing HDL particles through inhibition of their uptake by hepatocytes. However, these particles, which can transfer a portion of their cholesterol content into the liver, are more effective in absorbing additional cholesterol from peripheral cells and, therefore, in promoting reverse cholesterol transport. In addition, niacin positively affects the distribution of favorable HDL subtypes. There are at least two ways in which niacin has been shown to reduce serum levels of triglycerides. First, niacin plays an important role in inhibiting lipolysis within adipose tissue through inhibition of hormone-sensitive lipase, leading to decreased synthesis of fatty acids and triglycerides. Second, niacin may inhibit an enzyme called hepatic diacylglycerol acyltransferase 2, which mediates synthesis of triglycerides that are specifically targeted to assembly of VLDL. This inhibition in production of VLDL may be one way in which niacin also lowers levels of LDL (12, 13).

Although niacin has been successfully used in management of dyslipidemia associated with type 2 diabetes and metabolic syndrome (14) as well as alcohol-induced lipemia (10), its effect on dyslipidemia and fatty liver due to GC administration have not been addressed yet. Regarding the especial mechanisms responsible for lipid disturbances due to GCs and the unique profile of resulted dyslipidemia (elevated levels of HDL-c), our study was conducted to clarify whether niacin can counteract this type of dyslipidemia. Moreover, possible effect of niacin on fatty changes of liver due to GC administration has been addressed in the present study. 

Mitamura 1987, observed a dose-dependent increase in cholesterol concentration of HDL_1_ and HDL_2_, but no significant change in apolipoprotein B-containing lipoprotein cholesterol levels in rats treated with triamcinolone for 5 days (4). Bagdade *et al* 1976 observed significant increases in triglycerides and VLDL triglyceride associated with no change in cholesterol and an actual reduction in both triglyceride and cholesterol in LDL in rats treated with dexamethasone for two weeks (9). In this study, special measures were under taken to maintain positive caloric balance. In the study performed by Cole *et al* 1982, dexamethasone administration for 7 days in rats, increased the concentration of plasma free fatty acids and triglycerides along with VLDL protein, triglyceride, phospholipid, and free cholesterol. No changes were observed in the concentration or composition of plasma LDL lipids. The concentration of plasma HDL protein and lipid tended to increase (15). In the present study, dexamethasone administration induced an obvious dyslipidemia which was demonstrated by elevations in serum levels of total cholesterol, triglycerides, LDL-c, HDL-c and VLDL-c. The partial discrepancies observed in serum lipid parameters among different studies may be due to different dosages, treatment periods, agents and the whole condition of the experiments. 

Treatment of rats with GCs has led to accumulation of lipids in the liver (15). In the present study dexamethasone induced moderate degrees of fatty liver in one third of dexamethasone treated rats which was not observed in dexamethasone+niacin treated rats. 

Regarding the lipid profile, niacin significantly lowered total cholesterol, triglycerides, LDL-c and VLDL-c levels as compared to dexamethasone treated rats.

The only serum lipid parameter of dexamethasone+niacin treated rats that reduced to the control level was total cholesterol. It seems that although niacin can extenuate dyslipidemia due to GC administration, it cannot completely reverse it to the normal values. Another interesting finding was the fact that the level of HDL-c in rats treated with dexamethasone+niacin remained higher than control group and statistically the same as rats treated with dexamethasone. This may be due to the intrinsic property of niacin for increasing HDL-c levels (14). 

## Conclusions

In conclusion our results demonstrate that niacin has positive effects on dyslipidemia and fatty changes of liver due to dexamethasone administration in rats. 
